# Intelligent Grapevine Disease Detection Using IoT Sensor Network

**DOI:** 10.3390/bioengineering10091021

**Published:** 2023-08-29

**Authors:** Mihaela Hnatiuc, Simona Ghita, Domnica Alpetri, Aurora Ranca, Victoria Artem, Ionica Dina, Mădălina Cosma, Mazin Abed Mohammed

**Affiliations:** 1 Electronic and Telecommunication Departament, Constanta Maritime University, 104 Mircea cel Batran, 900663 Constanta, Romania; 2 Murfatlar Research Station for Viticulture and Enology, 905100 Murfatlar, Romaniaartemv@statiuneamurfatlar.ro (V.A.); dinai@statiuneamurfatlar.ro (I.D.);; 3 College of Computer Science and Information Technology, University of Anbar, Ramadi 31001, Iraq

**Keywords:** prediction algorithm, data correlation, IoT system, sensors, disease identification

## Abstract

The Internet of Things (IoT) has gained significance in agriculture, using remote sensing and machine learning to help farmers make high-precision management decisions. This technology can be applied in viticulture, making it possible to monitor disease occurrence and prevent them automatically. The study aims to achieve an intelligent grapevine disease detection method, using an IoT sensor network that collects environmental and plant-related data. The focus of this study is the identification of the main parameters which provide early information regarding the grapevine’s health. An overview of the sensor network, architecture, and components is provided in this paper. The IoT sensors system is deployed in the experimental plots located within the plantations of the Research Station for Viticulture and Enology (SDV) in Murfatlar, Romania. Classical methods for disease identification are applied in the field as well, in order to compare them with the sensor data, thus improving the algorithm for grapevine disease identification. The data from the sensors are analyzed using Machine Learning (ML) algorithms and correlated with the results obtained using classical methods in order to identify and predict grapevine diseases. The results of the disease occurrence are presented along with the corresponding environmental parameters. The error of the classification system, which uses a feedforward neural network, is 0.05. This study will be continued with the results obtained from the IoT sensors tested in vineyards located in other regions.

## 1. Introduction

The Internet of Things (IoT) technology has greatly developed in recent years, succeeding in replacing human labor by monitoring from a distance using certain devices. IoT devices collect information on various environmental conditions; this way, the farmer gains the advantage of accessing crop data without being present in the field. A new concept of Artificial Intelligence of Things (AIoT) has been developed to help accelerate the development of field monitoring and the identification of crop issues [1]. While reducing management costs, IoT technology can improve productivity by minimizing production loss through an early and accurate diagnosis [2]. Wireless Sensor Networks (WSNs) are a distance communication technology that are prominently used in intelligent farms [3]. Using this technology, the data from the sensors placed on the field are transmitted to winegrowers [4].

Disease detection is an intensive area of research in viticulture. They are caused by fungi or bacteria. The main grape diseases caused by fungi are downy mildew, powdery mildew, and black rot. Early disease identification can have a significant impact on yield and economic costs for the farmer. Plant growth and development, as well as disease severity, are directly affected by heat stress resulting from temperature changes. When pathogen-susceptible plants are grown in areas with frequent temperature changes, different pathogens are able to cause disease because they can withstand a wide range of environmental variations. The studies on grapevine disease prevention have many studies using IoT meteorological data. Being used for a variety of applications, IoT technology takes on a multitude of shapes and structures for each of them. The first separation is the one related to the Internet part and the object part. Furthermore, following this separation, a series of layers are defined, from 3 to 7. In its simplest version, the IoT architecture consists of three layers: the perception layer, network layer, and application layer [5,6,7]. Increased humidity triggers certain diseases, progressing mainly by altering the infection process, spore germination, and spore dissemination. Rain and high humidity trigger the infection of aerial plant tissues by pathogens. Relatively high atmospheric humidity (>85%) is favorable for the appearance of fungi, bacteria, and the development of diseases. For most fungal pathogens, leaf wetness (the length of time the leaf has water on its surface) is critical for disease development. High atmospheric humidity influences resistance to invasion by Botrytis cinerea and Penicillium expansum. Traditional monitoring of grapevine diseases involves visual assessments performed by specialists. In addition to being subjective, visual evaluation has the disadvantage of being labor-intensive, especially in the case of large plantations [8].

Environmental factors influence the growth of plants, which can be observed in the stems, leaves, fruits, and flowers. IoT sensor systems are designed to acquire data about environmental factors and plants. Data acquired from sensors are analyzed with adaptive algorithms in order to ensure better management strategies in precision viticulture [9,10,11]. Automatic data collection allows for permanent crop status observation. By applying treatments after identifying the onset of diseases, the spread of the infection and the excessive use of pesticides can be reduced, thus having a lower long-term impact on environmental, animal, and human health. Light is the most important environmental factor for circadian regulation. Plasmopara viticola, which causes grapevine downy mildew, the immature form of sporangia has been observed in continuous light and has no effect on sporangia formation and mycelial growth [12].

A hybrid of Support Vector Machine (SVM) and Logistic Regression (LR) algorithms were described in another IoT crop monitoring study [13] to predict powdery mildew disease in tomato plants. Adaptive Sampling (ANR) was applied to minimize the noise in the data, using the filtering method. The resulting training set obtained from the SVM-ANR method was further fed into the LR classifier to develop the classification model. The proposed SVM-LR hybrid method achieved higher accuracy in powdery mildew disease prediction compared to the SVM and LR algorithms alone. However, the paper did not use any feature selection algorithm to identify the most important features. Another study [14] presents a set of fungal disease models applicable to viticulture. Using meteorological variables, diseases like downy mildew, powdery mildew, and black rot are monitored. The detection models have been adapted to be learning in real-time and work with IoT SEnviro nodes [15], which are capable of generating information at adaptive levels. The IoT nodes are deployed in a vineyard in the province of Castelló on Merlot, Monanstrell, Bonicaire, and Cabernet (Spain). These sensors stored the data for 130 days (May–September 2018). Using the threshold for the data of the parameters that can produce the disease, an alarm is sent on the website in case of disease occurrence. The described methods are proposed for the reduction in phytosanitary products. Another study [16] correlates the data collected with the classical method on leaf stomatal conductance and data from the SF-4 Micro Stem Sap Flow Sensor. This method is used for calibration slopes for three different plants (a) *Hakea* sp. (R2 = 0.90), (b) *Ardisia* sp. (R2 = 0.76), and (c) *Fragaria* sp. (R2 = 0.82), where *p* < 0.05. The comparison between the sap flow data of the SF-4 sensor and the volume of water measured with classical methods is highly significant (R2 = 0.95). This shows that distance monitoring gives good results using sensors implemented on the field.

The presented work was developed in a Romanian vineyard, where the environmental parameters were different in comparison to Spain, France, or Greece [15]. The designed IoT system and algorithm will be tested in other vineyards to validate the model. The main objectives of this study are:

- To identify the degree of attack on the leaves using classical methods;
- To deploy the sensors in the vineyard;
- To correlate the sensors data with data collected using classical methods;
- To cluster the data from the sensors to detect the numbers of diseases that can be predicted with data from sensors;
- To develop an algorithm for the automated detection of grapevine disease.

As a goal, the present study aims to detect diseases at the point when their evolution can still be stopped, using a prediction algorithm and comparing the data taken from IoT sensors with the data obtained using classical methods, in order to verify the accuracy of sensor data.

This paper is structured in five sections. Section 2 presents the materials and the methods used for classical and IoT grapevine monitoring, as well as data processing. Section 3 describes the results obtained from data analysis and future work and recommendations. The discussions are presented in Section 4. The last section offers the conclusions and future research recommendations.

## 2. Materials and Methods

### 2.1. Plant Material and Classical Data Collection Methods

Data acquisition was carried out in field conditions in the plantations of SDV, Murfatlar. Two grapevine cultivars were chosen to be monitored using classical and sensor-based methods: Sauvignon Blanc and Cabernet Sauvignon. For each type of vine, a treated and untreated plot was established. For each variant, 15 grapevine plants were kept under observation using classical methods, and one plant was chosen to be monitored using sensors. The active vegetation period was 148 days for Sauvignon Blanc and 151 days for Cabernet Sauvignon during the year 2021. An agronomic protocol was elaborated in order to gather information on the health of the grapevine, which included disease monitoring and plant physiology determinations, namely, stomatal conductance and leaf relative chlorophyll content. In each plot during the vegetative season, the development of the following three grapevine diseases was monitored: downy mildew, caused by Plasmopara Viticola; powdery mildew, caused by Uncinula necator; and grey rot, caused by Botrytis cinerea. Downy mildew develops especially in conditions of high humidity and moderate temperatures, usually between 12–25 degrees Celsius, a relative air humidity of 92–100%, and a leaf humidity of 24% (Table 1). For gray rot, symptoms include the appearance of a gray mold layer on the surface of plants, loss of color and texture, and rotting and drying of fruits and inflorescences. In case of severe infection, the plant may die completely. The fungus develops especially in conditions of high humidity and temperatures between 18–20 degrees Celsius and humidity 80–100% and leaf humidity 72–90%. Symptoms of the disease include yellowish or green spots on the vine leaves, which later turn into brown or black spots.

In order to follow the phytosanitary condition of the grapevine, the vegetation phenophases of the two studied varieties were taken into account, according to the BBCH scale (Biologische Bundesanstalt Bundessortenamt and Chemical Industry, a universal scale for plant phenology, where each phenological stage is noted with numbers) [9], along with the biology of the pathogen and the climatic factors. Considering the fact that the foliar apparatus is the indicator of the phytosanitary condition of the plant, the leaf was the vegetative organ used for disease assessment for the classical method. The extent of the diseases was assessed using a visual graphical scale [14]. Disease assessments were performed according to the degree of attack (DA). This value represented the extent of the attack on the crop reported by the total number of plants on which the observations were made; results after calculating the frequency of the attack was F; and the intensity of the attack was I, as follows (1) and (2):

(1) $$GA=\frac{F\times I}{100}$$

(2) $$F=\frac{N\times 100}{Nt}$$

*N*: the number of attacked plants (leaf);

*Nt*: the total number of observed plants (leaf).

The value of the frequency of the attack informs us only about the spread of the infected area.

*I*: the intensity of the attack.

Chlorophyll content and stomatal conductance are relevant indicators of a plant’s physiological state [17]. The stomatal conductance was measured using a steady-state porometer, and chlorophyll measurements were achieved with SPAD 502 Plus chlorophyllmeter. For each variant, 10 leaves were analyzed, measuring values on 10 distinct points for each of them. The field analyses were performed as follows: BBCH 11–19 (30.04–17.05) for downy mildew, BBCH 69–79 (14.06–30.07) for powdery mildew, and BBCH 79–85 (30.07–20.08) for gray rot. For each disease, the aforementioned periods corresponded to the theoretical onset of symptoms, given the optimal environmental conditions were identified.

### 2.2. Methods and Algorithms

#### 2.2.1. Clustering Methods

The evaluation of the algorithm’s performance was achieve using four parameters: inertia; Silhouette coefficient; Calinski–Harabasz index; and the Davies–Bouldin index. Inertia measured how well a data set was clustered by the K-Means algorithm. It was calculated by measuring the distance between each data point and its centroid, squaring this distance, and summing these squares for each cluster (Formula (3)).

(3) $$\overset{N}{\underset{i=1}{\sum }}{\left(x_{i}-C_{k}\right)}^{2}\;$$

*X*_i_: data point;

*C_k_*: centroid;

*I*: data number;

*K*: cluster number;

*N*: maximum data number.

A good model is one with low inertia and a small number of clusters (K). However, this is a trade-off since as K increases, inertia decreases.

The Silhouette coefficient is used to assess the quality of clusters created by clustering algorithms such as K-Means, based on how well similar samples are grouped together. The Silhouette coefficient is calculated for each sample in a cluster. To determine the Silhouette coefficient for each observation/data point, the following distances must be found: average distance between the observation and all other data points in that cluster. This distance can also be called the average intra-cluster distance. Average distance between the observation and all other data points in the next closest cluster. This distance can also be called the average distance between the closest clusters.

The variation range of this parameter is [–1, 1]. If it is 1, the cluster is dense and well separated from other clusters; if it is 0 or close to 0, it means there are overlapping clusters or samples that are very close to the decision limit of neighboring clusters. If the score is in the range [−1, 0], it means that there are samples assigned to the wrong clusters [18].

The Calinski–Harabasz (CH) index is used to evaluate the model when the ground truth is not known, where validation of how the clustering was achieved is performed using quantities and characteristics inherent to the data set.

The CH index provides information on the similarity between an object and its own cluster—cohesion—and in comparison with other clusters—separation. Here, cohesion is estimated based on the distances between the data points in a cluster and the centroid, and separation is based on the distance between the centroids of each cluster and the global centroid. The CH index has the form (a.separation)/(b.cohesion), where a and b are weights.

The CH index for a number k of clusters on a data set D. Where D is d_1_, d_2_, and d_3_, …, and d_N_ defined as follows (4):

(4) $$\mathrm{CH}=\left[\frac{\sum_{k=1}^{K}n_{k}{\|c}_{k}-{c\|}^{2}}{K-1}\right]/\left[\frac{\sum_{k=1}^{K}\sum_{i=1}^{n_{k}}{\|d}_{i}-c_{k}{\|}^{2}}{N-K}\right]$$

*n_k_*: points number;

*c_k_*: centroid k clusters;

*c:* global centroid;

*N*: total number of points of data.

A high value of the CH index represents dense and well-separated clusters. In this case, there is no threshold value. The Davies–Bouldin Index (DBI) is one of the evaluation measures of clustering algorithms. It most commonly used to evaluate the quality of the split performed by a K-Means algorithm for a given number of clusters. The DBI is computed as the average similarity of each cluster to its most similar cluster. If the average similarity is low, the clusters are well separated, and the result of the clustering is good.

#### 2.2.2. Classification Methods

Decision trees (DTs) are a supervised learning technique used for classification and regression problems. Generally, DT are used for solving classification problems. This algorithm is a classifier structured in the form of a tree, where the internal nodes represent the features of the data set. The branches represent the decision rules, and each leaf node represents the output. In a decision tree, there are two types of nodes: decision node and leaf node. Decision nodes are used to make decisions and have multiple branches, whereas leaf nodes are the result of these decisions and contain no further branches. Decisions or tests are based on the features in the analyzed data set.

To build a tree, we used the CART algorithm (Classification and Regression Tree Algorithm). A question is asked, and, based on the answer (Yes/No), the tree is further divided into subtrees. The disadvantage of a DT is its multiple layers, which lead to a complex algorithm. If the algorithm is multiclass, this situation is accentuated. Too much complexity can lead to the phenomenon of “overfitting” (the formed/found hypothesis includes noise or irrelevant data patterns), but this problem can be solved using the Random Forest algorithm.

Random Forest (RF) is a machine learning algorithm that produces a good result in many cases, even without hyper-parameter tuning. It is also one of the most widely used algorithms due to its simplicity and versatility of use. Like DT, it is used for both classification and regression problems.

The confusion matrix is an N × N matrix applied in order to determine the performance of a classification model, N being the number of targeted classes. Thus, the target values are compared with the values predicted using the machine learning model, obtaining a whole-picture view on the performance of the model and the potential errors that might result.

Accuracy, Recall, Precision, and F1 Scores are used for performance of a model.

Accuracy is the intuitive measure of performance, the ratio of the observations predicted correctly, and the total number of observations.

- Precision is the ratio of correctly predicted positive observations to the total predicted positive observations (5).

(5) $$precision=\frac{TP}{TP+FP}$$

*TP*: number of true positive cases;

*FP*: number of false positive cases.

- Recall is the ratio of correctly predicted positive observations to the total observations in the class (6).

(6) $$Recall=\frac{TP}{TP+FN}$$

*FN* = number of false negative cases.

- *F1*-Measure takes precision into consideration as well as recall, thus analyzing false-negative and false-positive values (7).

(7) $$F_{1}=\frac{TP}{TP+\frac{FN+FP}{2}}$$

A classification method can be achieved using a neural network (NN). A feedforward neural network can be composed of three types of nodes:

- Input nodes: this provides the network with information from the outside world, and all input nodes form the input layer together;
- Hidden Nodes: they have no direct connection to the outside world. They perform calculations and transfer information from input nodes to output nodes;
- Output nodes: these are responsible for computations and transferring information from the network to the outside world.

The algorithm for data clustering and classification is used in this study [18,19,20,21].

### 2.3. Data Acquisition Using IoT Technology

**Figure 1:**
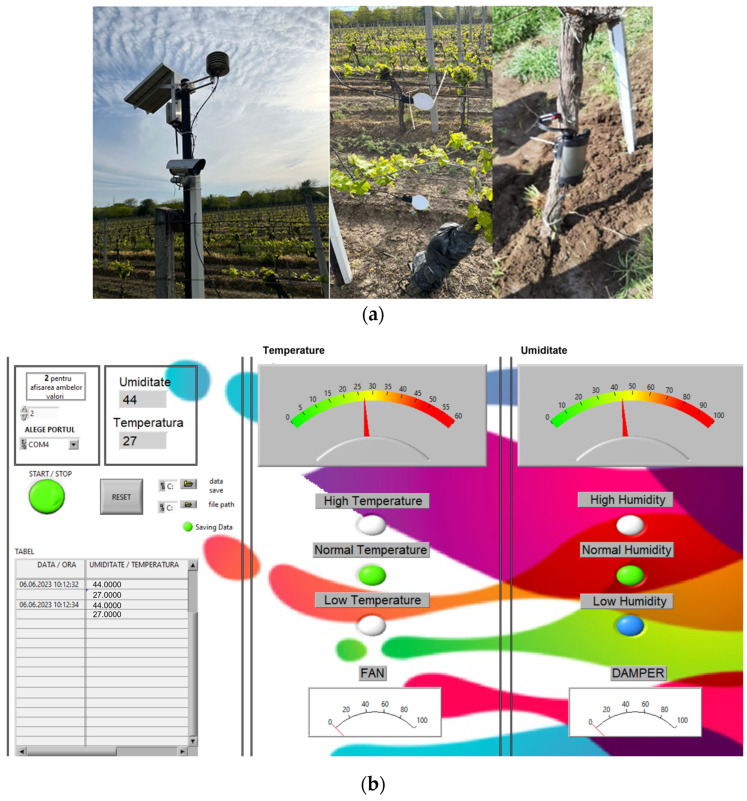
(**a**) The sensors network implemented on the vineyard Murfatlar; (**b**) the interface designed in LabView, used for data presentation and analysis from the sensors.

The experimental system consists of sensor kits based on IoT technology, in concordance with the classical method. The IoT network has the following components: sap flow meter; air temperature and humidity sensor; solar radiation sensor (PAR); leaf humidity sensor; soil temperature and humidity sensor; and soil oxygen sensor. The sensors were installed on the field in May 2021 at the beginning of the grapevine growing period (Figure 1a).

Two solar panels of 10 W and one solar panel of 20 W were used for powering the sensors and charging the energy sources, as the producer proposed [22]. Data were transmitted to the cloud using MicroGateway RAK7258. The original cloud was Azure. The transmission of Wi-Fi (Wireless Internet Frequent Interface) data was performed through the LORAWan-EU868 protocol. The data transmission distance between the node and gateway was in the range of 100 m–2 km. There were two nodes with sensors, one for the Sauvignon Blanc plot and the other for the Cabernet Sauvignon plot. The data from the sensors were recorded on an 8 GB external memory card.

Data could be graphically viewed in the Human Machine Interface (HMI) designed in LabView from National Instrument or as a table (*.csv format) in order to correlate the results (Figure 1b). The interface showed the values of temperature, humidity, atmospheric pressure, and wind direction. Depending on the thresholds written in the code, the possibility of downy mildew and gray rot could be identified. These parameters influenced disease occurrence. The favorable atmospheric conditions for disease occurrence were included in the interface code. The code obtained in LabView used the thresholds of the main parameters air temperature and humidity.

Data signals from the sensors were analog (with voltage or current output) or digital (SDI-12 protocol). The IoT system was configured by the company ICT International from Australia. The sensors used in the study were:

(a) Leaf moisture—PHYTOS 311 type, designed with thin fiberglass. This sensor is dielectric, with an output voltage of [320; 1000] mV and a 3 V power supply. The sensors work in the temperature range of −30 °C and +40 °C;
(b) Soil O_2_-SO-411 type has a 12 V power supply and works in the range of [−10; +50] degrees Celsius;
(c) Moisture and temperature—SDI-12 type, has a digital output type, with a supply voltage of 12 V. The measuring range of humidity is 0–60% and temperature in the range of [−30; +70] °C. The sensors are placed at a depth of 20 cm;
(d) Photosynthetically active radiation (PAR)-SQ-521 type is a digital sensor with a measurement range of [400; 700] nm;
(e) Air humidity and temperature digital sensor has a temperature range of [−30 °C; + 50 °C] and an air humidity range of [0%; 100%]. It is powered to 12 V;
(f) The SFM1 Sap Flow Meter measures the speed of sap flow in the stem.

**Figure 2:**
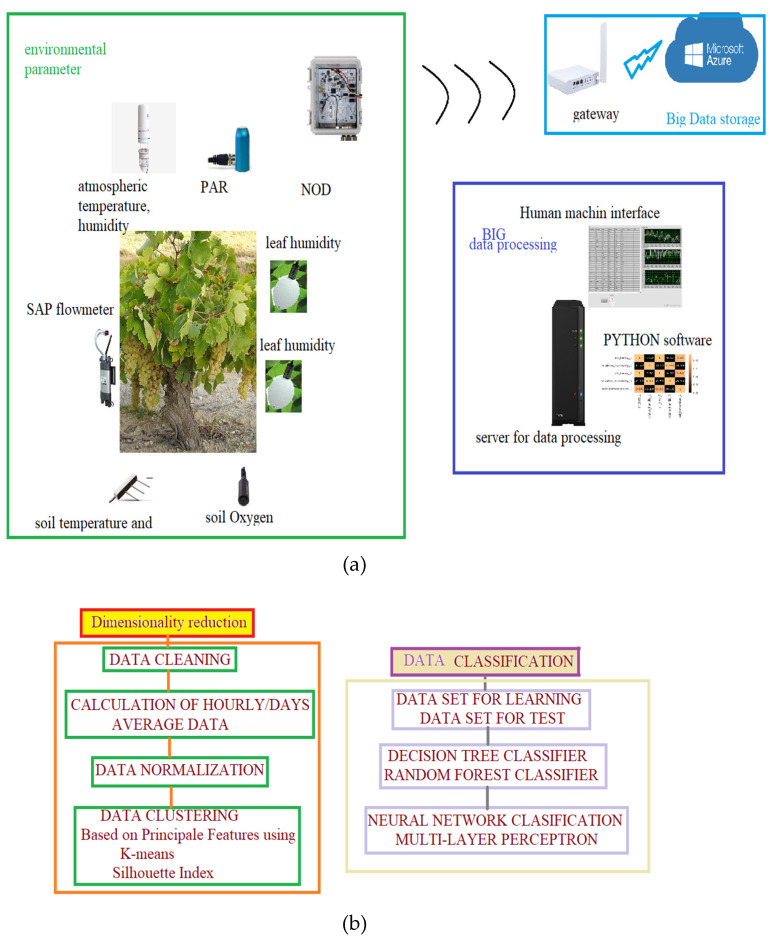
(**a**) Bloc diagram of data transmission, acquisition, and processing; (**b**) data processing diagram.

A bloc diagram of the acquisition and communication system is presented in Figure 2a.

### 2.4. Data Processing

Environmental conditions and plant behavior were analyzed using the data collected from sensors, in concordance with the results obtained by classical methods. Data were analyzed in the Python programming language, using the open source Visual Studio Code development environment. The prediction was achieved using TensorFlow open sourse platform for ML (https://www.tensorflow.org/learn) (accessed on 24 July 2023). The computing device was a computer equipped with a Nvidia RTX 3090 video card with 24 GB GDDR6X, an 3.7 GHz Intel Core I9-10900x microprocessor, and 64 GB RAM. The operating system was Microsoft Windows 10 Pro. [23].

The data from each sensor were placed in a file and then read automatically for feature extraction. The correlation of data features was performed in order to identify the influences of the parameters on each other (Figure 2b). For variable analysis, the average values per hour were computed, the correlation of the normalized data was used, and, finally, a feedforward neural network was created to classify the data in order to predict the diseases chosen in the study.

## 3. Results

### 3.1. Disease Monitoring Using Classical Methods

**Figure 3:**
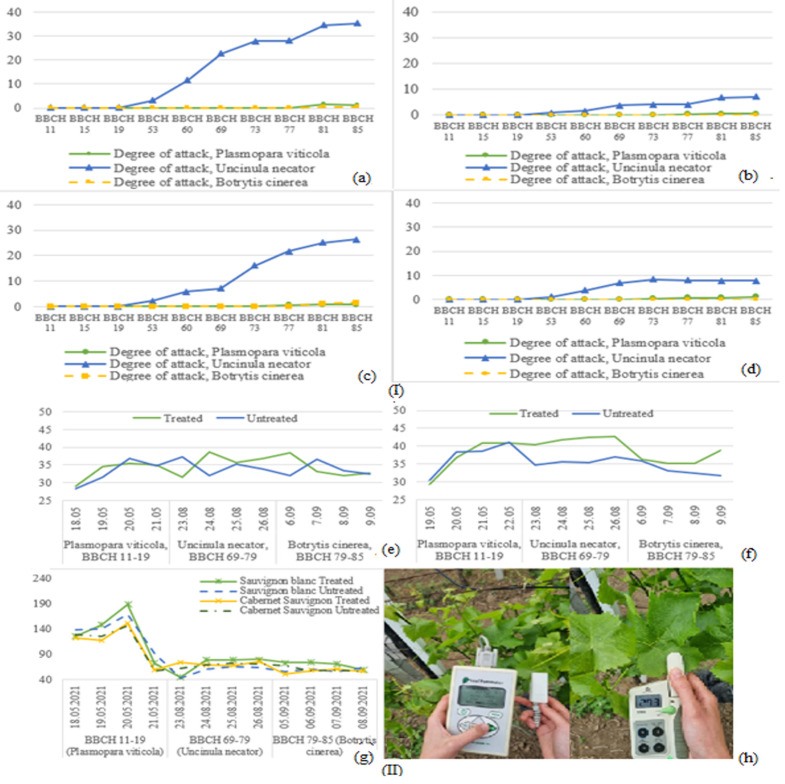
Degree of attack; (**a**)—Sauvignon blanc, untreated; (**b**)—Sauvignon blanc, treated; (**c**)—Cabernet Sauvignon, untreated; (**d**)—Cabernet Sauvignon, treated (**I**). Plant physiology analyses: Chlorophyll content, Cabernet Sauvignon; (**e**) Chlorophyll content, Sauvignon blanc; (**f**) Stomatal conductance for the studied plots; (**g**) Measuring chlorophyll with a SPAD 502 plus chlorophyll meter; (**h**) Measuring stomatal conductance with a Steady State porometer (**II**).

Out of the three studied diseases, only powdery mildew was present to a greater extent, registering a significant degree of attack at the foliar level for both cultivars (Figure 3(I)). The other diseases measured very low attack values in the periods designated for their observations. For the treated variants, the attack degree values were considerably lower, due to the application of phytosanitary treatments.

### 3.2. Plant Physiology Determinations

For Cabernet Sauvignon, similar chlorophyll content values were obtained for both variants during the measuring period for downy mildew, with higher values being recorded for the treated variant during the measuring periods for powdery mildew and gray rot (Figure 3(II)).

The lower chlorophyll values were mainly caused by the depigmentation of the leaf, due to the attack of the pathogens. The same observation could not be made for Sauvignon Blanc, where chlorophyll values were oscillating [24]. In regard to stomatal conductance, higher values are obtained when the plant is healthy, during BBCH 11–19. When the plant began to show symptoms of powdery mildew, during BBCH 69–79, the stomatal conductance significantly lowered for all the studied varieties. Similar results, where stomatal conductance registered lower values when the plant was infected with Uncinula necator, were obtained in another study [24,25].

### 3.3. Data Analysis

The correlated data using the maximum value could be interpreted as follows: PAR did not influence other parameters measured in either two of the correlations; SAP was influenced by both air and soil parameters, but not by leaf moisture. The data on leaf humidity in the two monitored areas were correlated in over 70% of cases and were influenced by the same environmental parameters: soil oxygen, moisture, and permeability, as well as air humidity, but only in the case of the maximum values. In order to improve the IoT monitoring system, a sensor for identifying the leaf temperature must be included so that a prediction of the disease could be made according to leaf and air moisture and temperature.

Leaf humidity values decreased, and the PAR value increased during the critical period of the appearance of downy mildew, thus not registering the optimal conditions for the appearance of this disease at the foliar level. Regarding sap flow measurements, the flow was periodic, not showing significant deformations, although disease symptoms were present. Starting with November, the plant began to reduce its activity, due to the onset of the dormancy period. As it is shown in the correlation diagram, sap flow is influenced by atmospheric conditions and soil parameters.

**Figure 4:**
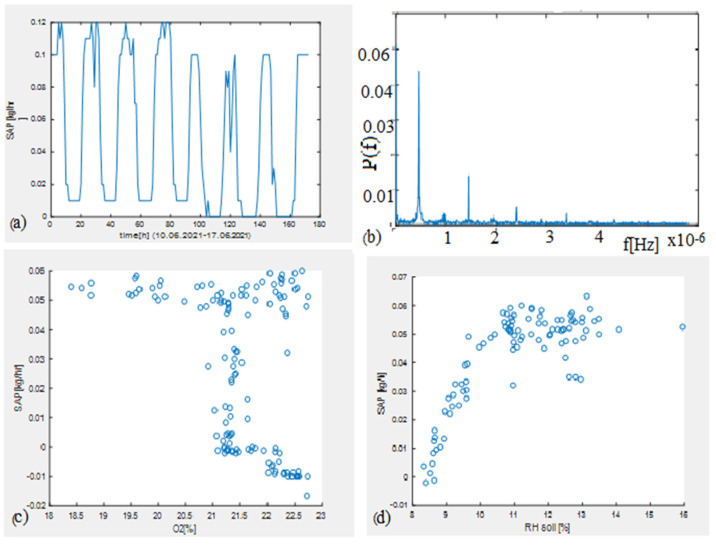
(**a**) The evolution of sap flow in 10–17 June 2021, (**b**) the FFT applied for SAP flowmeter signal during the vegetation period, (**c**) the sap flow function of the soil oxygen during the vegetation period, and (**d**) the sap flow variation function of the soil humidity during the vegetation period.

In order to perform correlation analyses, the chosen data interval was June–October. The characteristics extracted from each sensor were normalized, and a correlation was made between the maximum and mean values for each day. The sap flow meter signal had a periodic variation during the vegetation period Figure 4a, which could be observed after the FFT (Fast Fourier Transformation) presented in Figure 4b. There were no oxygen variations in the soil; therefore, sap flow variation was not present either (Figure 4c). After an increase in soil moisture of over 11%, there was a stabilization of the sap flow through the plant, even if the humidity increased (Figure 4d).

**Figure 5:**
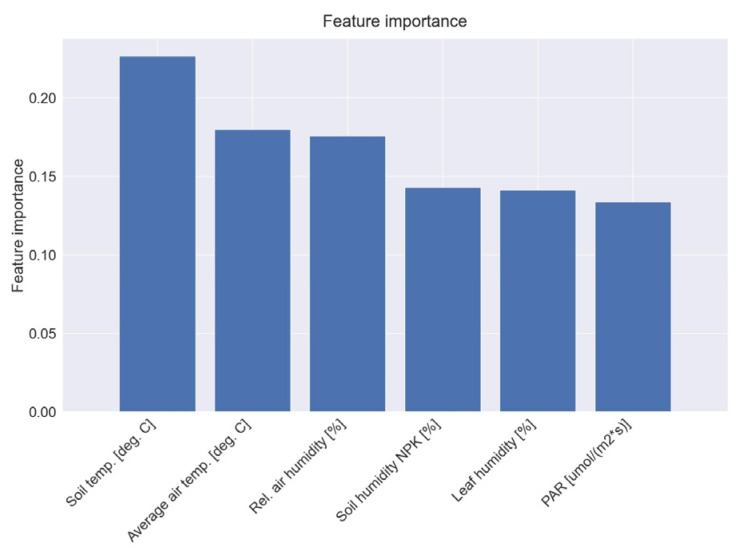
The feature importance for the environmental parameters data.

Using feature importance, we can identify the most significant parameters, which are then used in the classification methods. The temperature and humidity of air and soil are the most important parameters that can influence the disease occurrence (Figure 5). This confirmed the observation achieved using classical methods.

The score obtained after the feature importance analysis was bigger than 0.15, so all the parameters could be used as input in the prediction algorithm. The next section presents the results of the prediction and classification algorithm used for the automatic system, which will be developed at the end of the project.

## 4. Discussion

The classical method results can be used in the prediction system. The data acquired from sensors is used as the input in the algorithms, and the degree of attack for different diseases is used as the target. As can be seen from the data stored by sensors, leaf color is influenced by air temperature and humidity. The analysis is applied to the data from the Cabernet Sauvignon untreated plot in the vegetation period. After clustering two parameters, air temperature and humidity, the result of Silhouette index is equal to 0.63 for the two classes, which represent healthy and infected leaves. After the classical methods study, it was observed that the temperature range of 20 °C–25 °C had an influence on infection occurrence at moderate relative air humidity values. The results of our study were presented in paper [25], in which we analyzed the data from air parameters using different methods two classes identified. Using a scatter representation, it was observed that the data were linearly separable; therefore, the K-Means algorithm was suitable for the segmentation of the set (Table 2). There was some scatter, mainly at the edges of the clusters, noticeable through the value of 834,418,465 for inertia, which measured how well a data set was clustered by the K-Means algorithm. The CH index was 27,863.24, and the DBI index was 0.491, showing the average similarity of each cluster with the most similar cluster.

In this part of the project, the target column was added to the data set, which presented information about the state of the plant: diseased or healthy. The disease type was not indicated as output value. The classification was achieved using Decision Tree, Random Forest, and K-Nearest Neighbors algorithms.

The K-fold cross-validation predictions were made on the test subsets [21]. In our study, K-fold cross-validation was performed on three subsets (Table 3). The value of accuracy was comparable across all three folds, which indicated a good generalization ability. The highest accuracy is obtained using R.F.

After computing the confusion matrix associated with the Decision Tree Classifier, it was observed that a percentage of only 0.648% of the total analyzed cases was misclassified.

**Figure 6:**
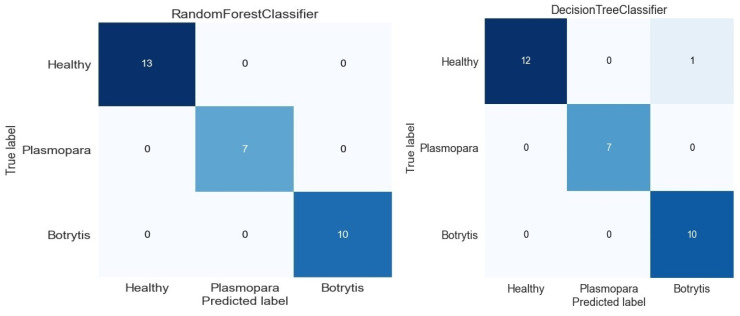
Confusion matrix for multiple parameters classifiers.

In the agronomic protocol, the disease type was indicated, as such, in the next step of the study, we predicted the Plasmopara and Botritis Cinera diseases. In Figure 6, the Confusion matrices associated with the three class classifications were presented. The data used in these analyses were soil and air humidity and temperature, soil oxygen, and PAR stored during the vegetation period of the 2022 year. This year, the disease was not frequently identified. So, using the confusion matrix, we could see that a good classification of the data in three groups was obtained. After the data classification for healthy and infected plants presented in [25], we could identify the disease type using more parameters in our analysis.

For the first set of data, classification was achieved using the DT and RF algorithms and only temperature and humidity as features. For the second data set, more features were included (Table 4).

For the first set of data, the results are presented in the next paragraph. The values of 1 for the negative class (the disease is not present) and 0.96 for the positive class (the disease is present) of the F1 parameter in the classification show good performance of the model. Comparing the ROC (Receiver Operating Characteristic) or PR (Precision/Recall) curve, the values of 5197 of all data, 506 indicate unbalanced classes. We use the Random Forest Classifier instead of the Decision Tree Classifier, which has many trees. This obtains performance and reduces overfitting. In the confusion matrix associated with the RF classifier, we have cases as 5190 TN, 487 TP, 19 FN, and 7 FFP. The total cases are 5703, out of which 5677 are correctly classified. In total, 0.45% of the total cases analyzed was misclassified. Here too, there is an improvement in comparison with previous cases, where wrongly analyzed cases represented 0.64% of the total (FN = 16, FP = 21).

Analyzing the F1 parameter, used in comparing the efficiency of the classifiers, it can be seen that the value 1 was still obtained for the negative class (the disease is not present), but there is an improvement in the result from 0.96 to 0.97 in the case of the positive class (the disease is present). Global accuracy also changed from 0.99 to 1.

In the second set of data improved for the RF classifier, the results are 1 in all the cases, and the global accuracy is 1. But, in the case of the DT classifier, the global accuracy is 0.97 less precise than in the first case.

After the classification algorithms presented above, we design a feedforward neural network (NN) to identify the disease. Using data collected from the field and from the sensors, a database is created, which is used to train a neural network. Daily maximum air temperature and humidity values from the interval April 2021–January 2022 are considered as inputs. These data are used because there are more data, and more infections are present in the field in the year 2021. All the input values are normalized in the range [−1, 1].

**Figure 7:**
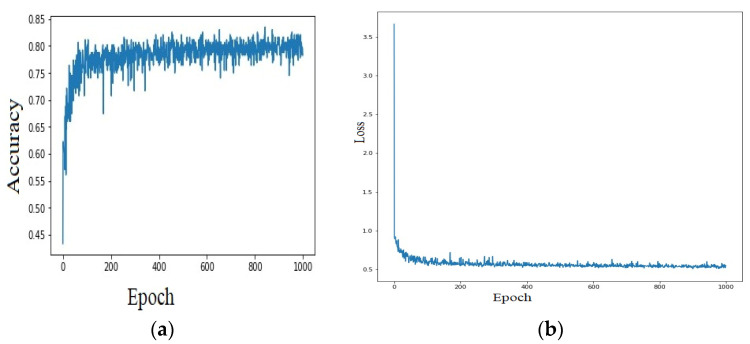
The neural network configuration for data classifications of (**a**) accuracy and (**b**) loss in predicting the occurrences of diseases using air temperature and humidity values.

The target output values are considered three classes with the labels: “0”—normal; “1”—Plasmopara; and “2”—Botritis. These diseases, which represent the NN outputs, are identified by classical methods, using the attack degree. The neural network has an input layer with 512 neurons and three hidden layers with 256, 128, and 64 neurons. All of these neurons have rectified linear (RELU) activation functions. In the output, there are three neurons with the SoftMax activation function. It is considered only three neurons, corresponding to the three classes. The neural network has dense layers, which means that each neuronal layer is closely connected to the previous. The 5197 data are used for training and testing NN. The NN is trained after 1000 epochs with 0.05 loss and an accuracy of over 85% (Figure 7a,b).

In this study, we analyze the different possibilities of disease identification using different environmental parameters. After the data correlation, we can observe as the temperature and humidity of the air influence the disease occurrence [26]. The present project has an advantage in the comparison between data collected from the sensors and using agronomic protocol [19,20]. In the NN classification, we use the target data obtained from the field regarding the degree of attack on the leaves collected in the vegetation period. In the future, we will analyze the data of an IoT sensor system built in the laboratory [22,27]. This system gives more information about the environmental parameters like soil nutrients, wind direction, leaf colors, and so on.

## 5. Conclusions

The main problem that can be solved with an IoT system is grapevine monitoring 24 h/day, creating the possibility of disease combat from a distance, without the intervention of farmers.

Comparing the conclusions of other articles that present the study in the same direction of plant disease detection, the results of this paper are very encouraging, obtaining accuracy values of approximately 0.95. As predictors, the main environmental parameters taken into consideration are air temperature and humidity. The robustness of the model is given by the data collected with classical methods. The difference between our study and others is the correlation between the results of classical and sensor monitoring methods. This paper is presented the accuracy of the intelligent models applied for different combinations of environmental parameters. On the dataset from the untreated Cabernet Sauvignon plot, DT classifiers are applied. The accuracy is 0.99. For the RF classifier, the accuracy is 0.97 for air temperature and humidity parameters. Very good results, but somewhat weaker than in the previous cases, are obtained with a particular NN algorithm, with an accuracy of 0.88.

However, more research is needed in order to allow a better performance of the elaborated new hyperparameters or architecture. Including the temperature and humidity of soil, the results of classification are very good, even 1 for the RF classifier. So, soil parameters have an important influence on disease occurrence.

The IoT sensor system will be improved with sensors that can give more information about soil environmental parameters. Automated methods will be designed to identify the disease and to prevent them using only the sensors. Another objective is to be able to identify more diseases using data sensors and algorithms.

In addition to the economic aspects, early grapevine disease detection helps to reduce the impact on the environment and on human health, as the number of pesticide applications is reduced.

## Figures and Tables

**Table 1:** The environmental conditions for the occurrence of the studied diseases.

Disease		Air Temperature [°C]	Air Humidity [%]	Leaf Humidity [%]
*Plasmopara viticola*	Occurrence	10	92–100	24
	Optimal	18–25	≥93	≥24
*Botrytis cinerea*	Occurrence	15	≥90	90
	Optimal	18–20	≥80	72–90
*Uncinula necator*	Occurrence	7–31	≥30	45
	Optimal	15	≥45	85

**Table 2:** The performance of the model [25].

Inertia	834,418.46
Silhouette Parameter	0.63
Index Calinski–Harabasz	27,863.24
Index Davies–Bouldin	0.491

**Table 3:** The predictions accuracy on the three test subsets K-fold cross comparing the DT with RF [25].

	Accuracy—Subset 1	Accuracy—Subset 2	Accuracy—Subset 3
Decision Tree (DT)	0.976	0.974	0.978
Random Forest (RF)	0.980	0.981	0.983

**Table 4:** Classification report for D T and R F Classifiers.

Classification Report: Decision Tree Classifier (two classes)				
	precision	recall	F1-score	support
0	1	1	1	5197
1	0.96	0.97	0.96	506
accuracy			0.99	5703
Macro average	0.98	0.98	0.98	5703
Weight average	0.99	0.99	0.99	5703
Classification Report: Random Forest Classifier (two classes)				
0	1	1	1	5197
1	0.99	0.96	0.97	506
accuracy			1	5703
Macro average	0.99	0.98	0.99	5703
Weight average	1	1	1	5703
Classification Report: Random Forest Classifier (multiple classifiers)				
	precision	recall f1	score support	
0	1.00	1.00	1.00	13
1	1.00	1.00	1.00	7
2	1.00	1.00	1.00	10
accuracy			1.00	30
Macro average	1.00	1.00	1.00	30
Weighted average	1.00	1.00	1.00	30
Classification Report: Decision Tree Classifier (multiple classifiers)				
	precision	recall f1	score support	
0	1.00	0.92	0.96	13
1	1.00	1.00	1.00	7
2	0.91	1.00	0.95	10
accuracy			0.97	30
Macro average	0.97	0.97	0.97	30
Weighted average	0.97	0.97	0.97	30

## Data Availability

https://cmu-edu.eu/meriavino-en/ (accessed on 24 July 2023).
